# Supplementary material for: Estimating hepatitis B virus cccDNA persistence in chronic infection
Source: Virus Evol. 2020 Aug 25;7(1):veaa063. doi: 10.1093/ve/veaa063 (PMC7947180; doi:10.1093/ve/veaa063)
Supplement: veaa063_Supplementary_Data [file veaa063_supplementary_data.zip › Supplementary Figures.docx]

**Estimating hepatitis B virus cccDNA persistence in chronic infection**

Katrina A. Lythgoe^1,2^, Sheila F. Lumley^3,4^, Lorenzo Pellis^5^, Jane A. McKeating^6+^ and Philippa C. Matthews^3,4,7+^

^+^ Denotes equal contribution

**Supplementary Figures**

**S1 Fig. Population and evolutionary dynamics of cccDNA for the within-host model assuming all cccDNA survives mitosis (*q*=1).** The model was parameterised assuming the generation time, *g*, during chronic HBeAg^POS^ infection is 61 days, and during chronic HBeAg^NEG^ infection is 26 days, in line with our predictions for cccDNA generation time *in vivo*. The top panel shows the cccDNA burden, where 1 represents the maximum possible burden. The middle panel shows the viral generation time (lines) and cccDNA lifespan during key stages of infection (dots, derived from Eqs 9 and 10). The bottom row shows the evolutionary rates. Black line: replicative capacity during HBeAg^NEG^ infection remains the same as during HBeAg-postive infection (*b_eAg+_= b_eAg-_*=0.3 per day). Blue line: replicative capacity falls to *b_eAg-_*=0.038 per day during HBeAg^NEG^ infection, and *R_0_*=1. Orange line: replicative capacity falls to *b_eAg-_*=0.038 per day and *R_0_*=0.7. See Table 1 for all other parameters.

**S2 Fig. Effect of NA treatment predicted by the model assuming all cccDNA survives mitosis (*q*=1).** A: cccDNA dynamics whilst on treatment, assuming some residual reproduction. For all cells *d*=0.002 per day, *δ*=0.014 per day, *c*=0, *q*=1. B: cccDNA dynamics on treatment, assuming no residual reproduction (*b*=0) but 0.1% cccDNA is long-lived, for different death rates of long-lived cells, *d_r_* per day. For normal cells *d*=0.002, *δ*=0.014, *c*=0, *q*=1, and for long-lived cells *δr*=0, *c_r_*=0, *q*=0. The maximum number of cccDNA was assumed to be 10^12^, and all model runs were started at equilibrium in the absence of treatment (*b*=0.3).
