# Supplementary figures and images for: Estimating hepatitis B virus cccDNA persistence in chronic infection
Source: Virus Evol. 2020 Aug 25;7(1):veaa063. doi: 10.1093/ve/veaa063 (PMC7947180; doi:10.1093/ve/veaa063)

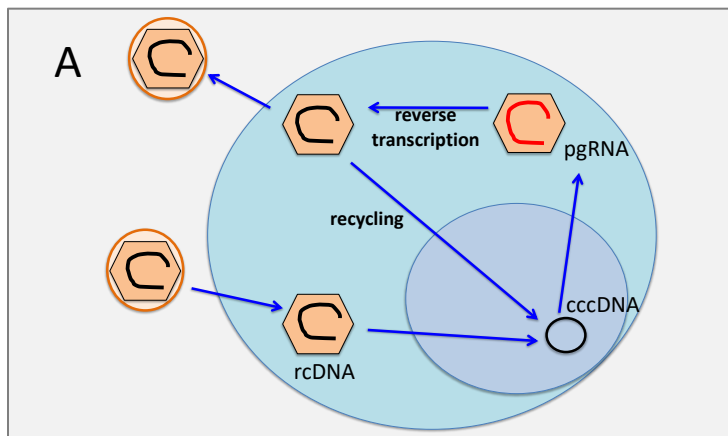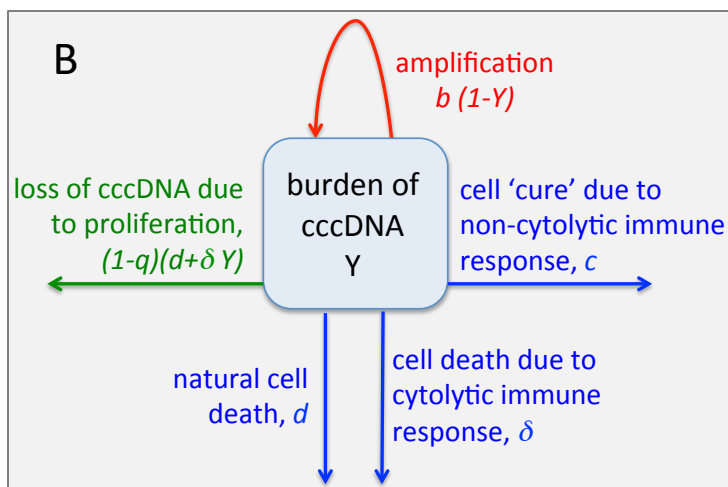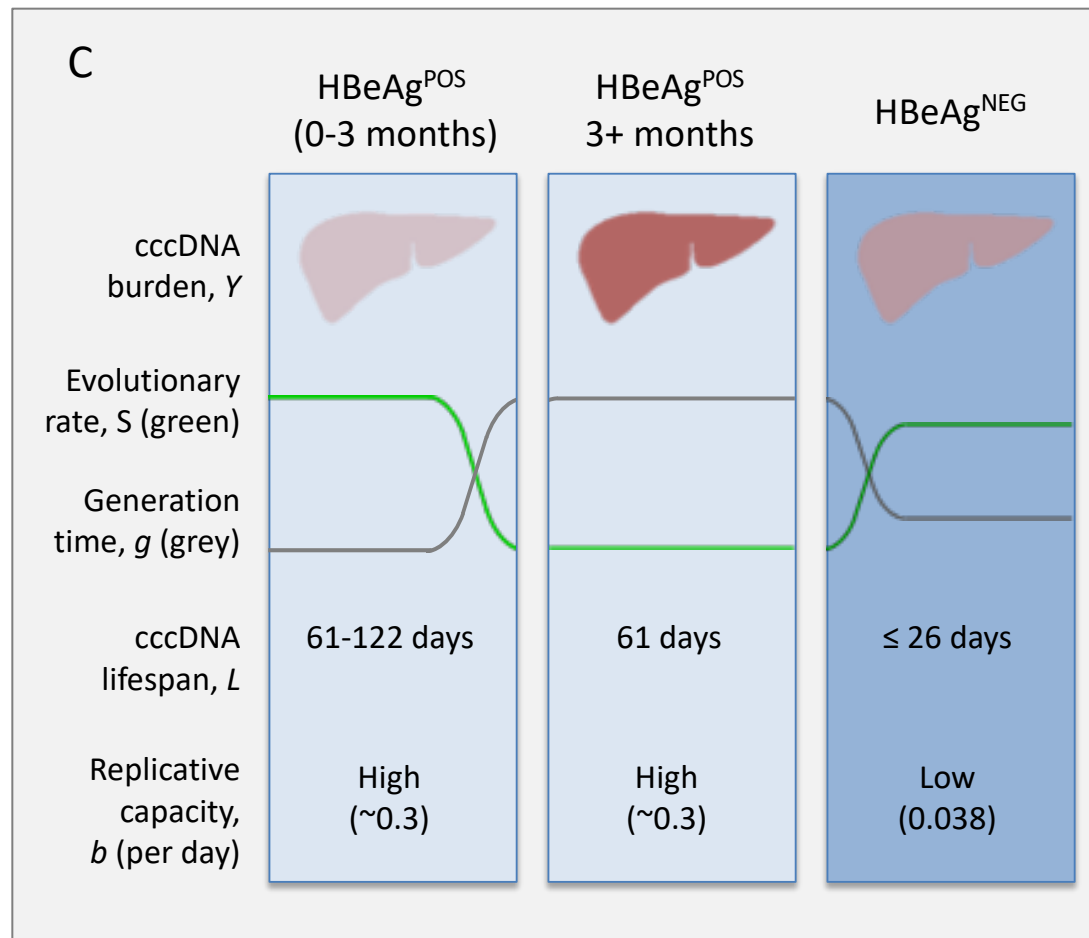

Supplement: veaa063_Supplementary_Data [file veaa063_supplementary_data.zip › OP-VEVO200064_PECorr_CmtAttachmentsFolder_Figure1_updated.pdf]
